# Anterior approach in situ resection for total laparoscopic pancreaticoduodenectomy

**DOI:** 10.1186/s12893-021-01433-7

**Published:** 2021-12-18

**Authors:** Yunqiang Cai, He Cai, Bing Peng

**Affiliations:** Department of Pancreatic Surgery, West China Hospital, Sichuan University, No. 37, Guo Xue Xiang, Chengdu, 610041 Sichuan China

**Keywords:** Laparoscopy, Pancreatectomy, Anterior approach

## Abstract

**Background:**

Laparoscopic pancreaticoduodenectomy (LPD) is gaining popularity in last decade. However, it is still technical challenging to perform LPD for patients with large periampullary tumors.

**Methods:**

From January 2019 to January 2020, 13 cases of LPD were performed via anterior approach. Data were collected prospectively in terms of demographic characteristics (age, gender, body mass index, pathological diagnosis and tumor size), intra-operative variables (operative time, estimated blood loss, transfusion), and post-operative variables (time for oral intake, post-operative hospital stay, and complications).

**Results:**

There were five male patients and eight female patients included in this study. The median age of these patients was 52.7 ± 14.5 years. The median size of tumors was 7.2 ± 2.9 cm. One patient converted to open surgery because of uncontrollable hemorrhage. The median operative time was 356 ± 47 min. The median estimated blood loss was 325 ± 216 ml. The mean post-operative hospital stay was 12.4 ± 1.9 days. One patient suffered from grade B pancreatic fistula. One patient suffered from delayed gastric emptying which was cured by conservative therapy. 90-day mortality was zero.

**Conclusions:**

Laparoscopic pancreaticoduodenectomy via anterior approach is safe and feasible for patients with large periampullary tumors. Its oncological benefit requires further investigation.

## Background

Laparoscopic pancreaticoduodenectomy (LPD) is gaining popularity because of improvements in surgical experience and technology [1]. However, it is still technical challenging to perform LPD for patients with large periampullary tumors. Traditionally, surgeons performed Kocher maneuver to complete mobilization of the duodenum and pancreas head before pancreas neck and uncinate of pancreas transection [2]. Unfortunately, performing Kocher maneuver for patients with large periampullary tumors is technically difficult because of its large size. Kocher maneuver also may increase the risks of tumor rupture and bleeding from the veins around tumor. Therefore, patients with a large tumor were considered to be unsuitable for LPD.

The caudal approach in laparoscopic hepatectomy, imitating the anterior approach in open procedure, has been regarded as one of the standard approaches in laparoscopic right hepatectomy [3, 4]. The anterior approach for open right hepatectomy can reduce the blood loss and operative time. Furthermore, it maybe associated with better oncological results by avoiding squeezing of tumor cells into the systemic circulation and avoids hepatic parenchymal tears [5]. However, laparoscopic pancreaticoduodenectomy via anterior approach was not described in the literature before. Herein, we reported 13 cases of LPD for large periampullary tumors via anterior approach and share our initial experience with this technique.

## Methods and materials

**Fig. 1:**
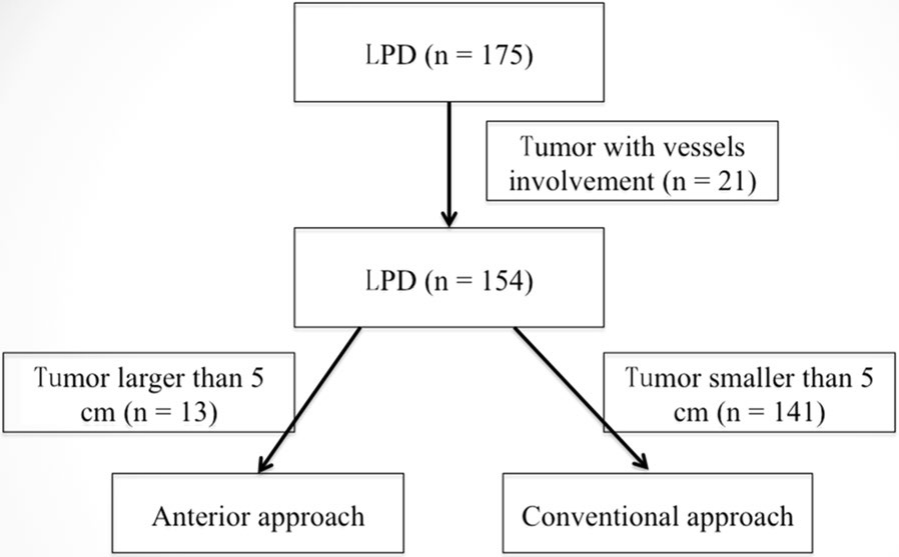
Flow diagram showing cases selected for laparoscopic pancreaticoduodenectomy via anterior approach. *LPD* laparoscopic pancreaticoduodenectomy

**Fig. 2:**
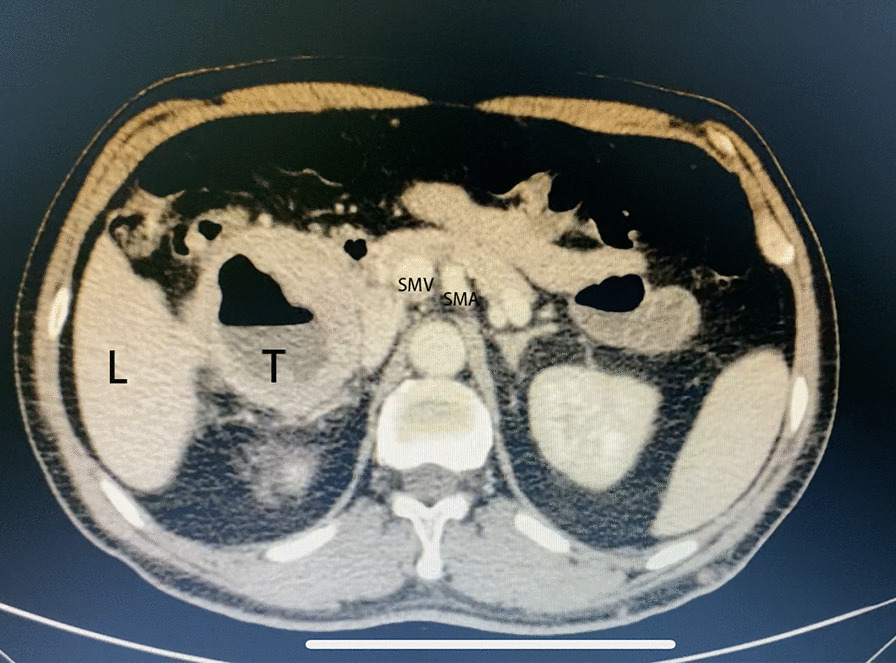
CT image of patient selected for laparoscopic pancreaticoduodenectomy via anterior approach. *L* Liver, *T* Tumor, *SMV* Superior mesenteric vein, *SMA* Superior mesenteric artery

From January 2019 to January 2020, we performed 175 cases of laparoscopic pancreaticoduodenectomy in our institution. All operations were performed by a single operative team. Among these cases, 13 cases of LPD were performed via anterior approach (Fig. 1). Generally, periampullary tumors larger than 5 cm without major vessels involvement or peripheral organ invasion were selected for anterior approach (Fig. 2). Data were collected prospectively in terms of demographic characteristics (age, gender, body mass index, pathological diagnosis and tumor size), intra-operative variables (operative time, estimated blood loss, transfusion), and post-operative variables (time for oral intake, post-operative hospital stay, and complications). Written consent was obtained from the patients associated in this study, and this study was permitted by the Ethics Committee of Sichuan University (WCH 2018-97).

## Operative procedure

### Patient’s position, trocar distribution

**Fig. 3:**
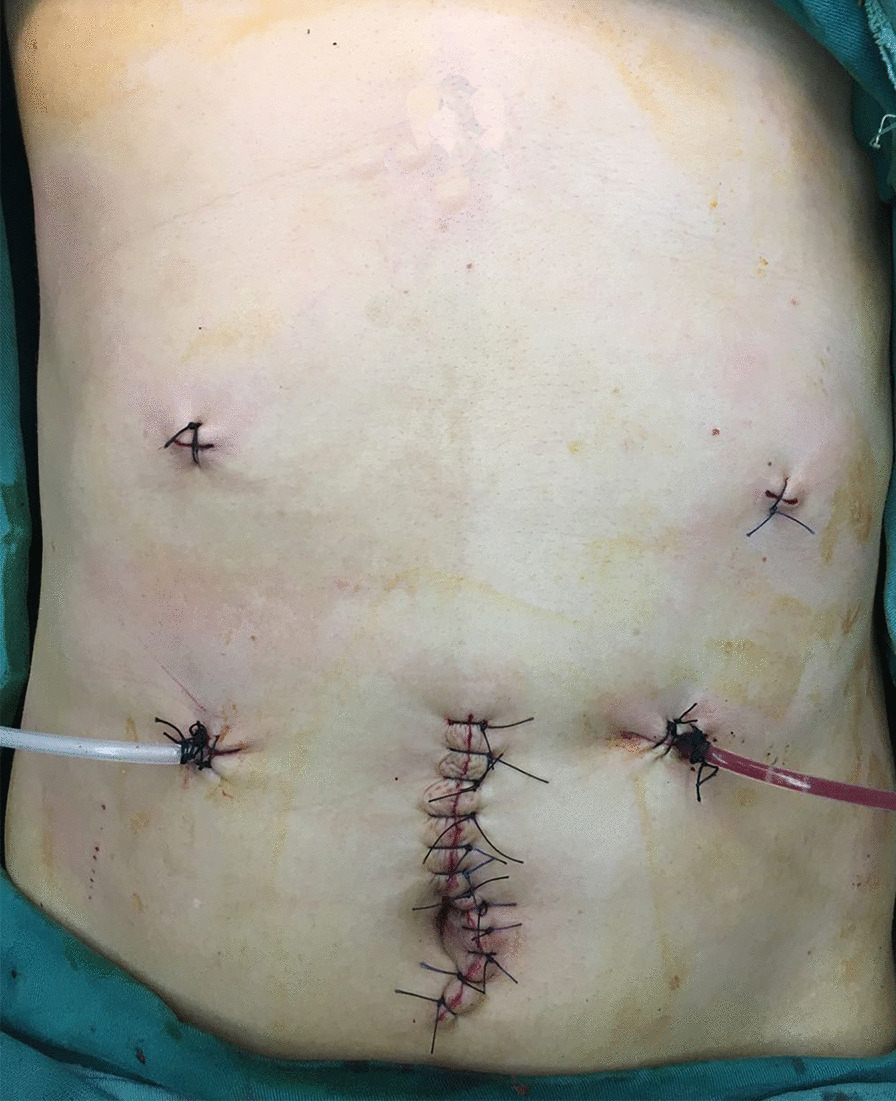
The trocars distributions

Patients were placed in supine position with two legs separated. Five trocars were used in all patients. General, the observing trocar (10-mm) was located at inferior umbilicus. The manipulating trocars (a 5-mm and a 12-mm trocar) distributed at right anterior axillary line and midclavicular line. The assistant trocars (two 12-mm trocars) distributed at left midclavicular line and anterior axillary line. The trocar distributions were shown in Fig. 3.

### Operative procedure

**Fig. 4:**
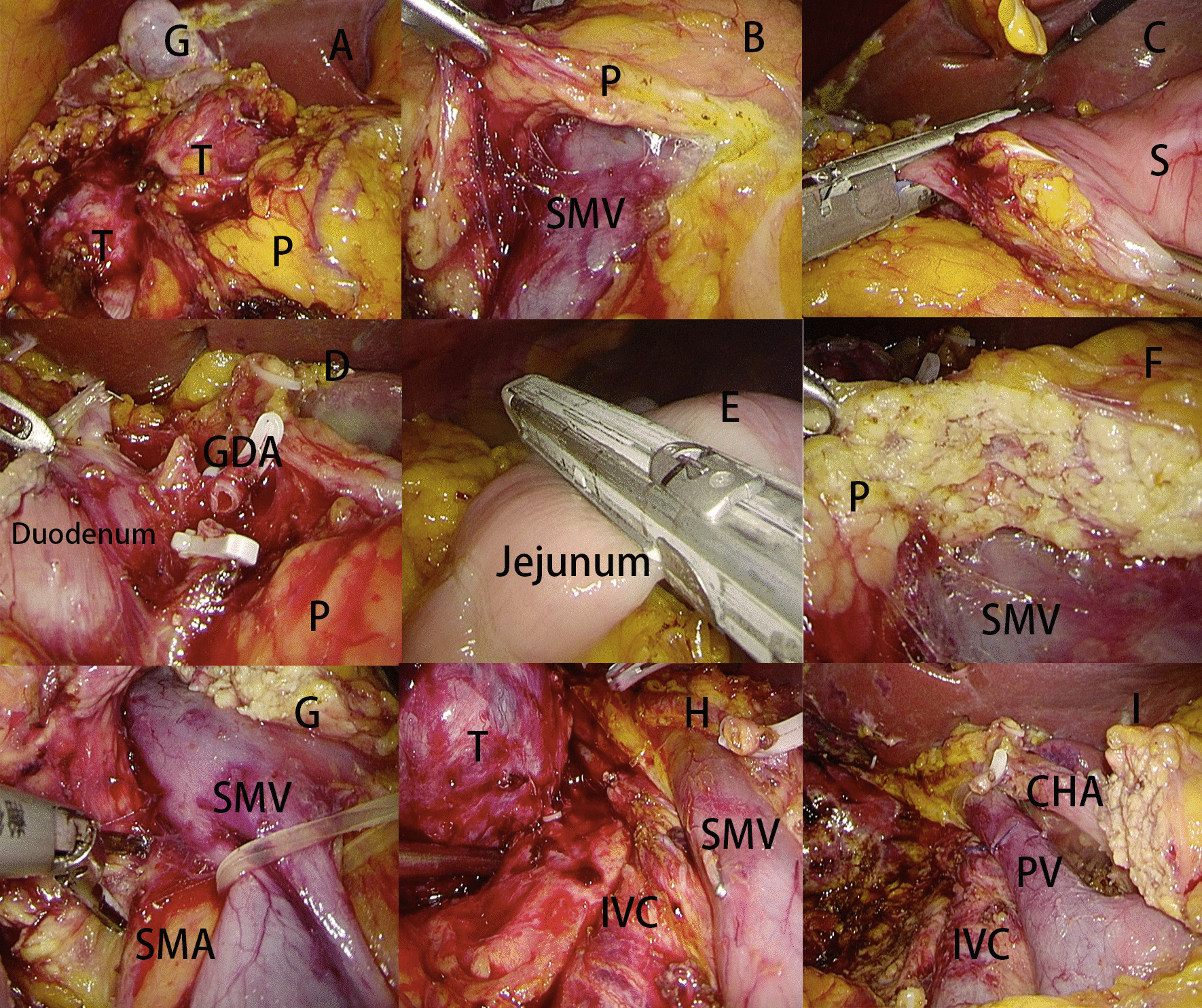
The main operative procedure of laparoscopic pancreaticoduodenectomy via anterior approach. **A** Tumor was shown after completely taking down the hepatic flexure of colon and mesocolon transverse; **B** Post-pancreas tunnel was created; **C** The duodenum was transected by endoscopic stapler; **D** The gastroduodenal artery was transected; **E** The jejunum was transected with endoscopic stapler; **F** The pancreas neck was transected by ultrasonic scalpel; **G** The uncinate process of pancreas was dissected at the right side of superior mesenteric artery; **H** Anti-Kocher maneuver was carried out; **I** The tumor was resected and lymphadenectomy was carried out. *T* Tumor, *P* Pancreas, *G* Gallbladder, *SMV* Superior mesenteric vein, *S* Stomach, *GDA* Gastroduodenal artery, *SMA* Superior mesenteric artery, *IVC* Inferior vena cava, *CHA* Common hepatic artery

The operation began with fully exploration of whole abdominal cavity. We dissected the omentum from left to right using ultrasonic scalpel. The hepatic flexure of colon and transverse mesocolon was taken down as fully as possible (Fig. 4A). The superior mesenteric vein (SMV) was identified at the low edge of pancreas neck and the post-pancreatic neck tunnel was created (Fig. 4B). Then the right gastroepiploic vessels and right gastric vessels were dissected. The duodenum/distal stomach was transected with endoscopic stapler (Fig. 4C). The No.8a lymph node was dissected and the common hepatic artery was identified and hanged with rubber band. The gastroduodenal artery was double clipped and transected (Fig. 4D). The gallbladder was dissected and the common hepatic duct was transected with scissors. The stump of common hepatic duct was clipped with bulldog clip in order to avoid bile juice contamination. Then the jejunum was transected with endoscopic stapler 15 cm from the ligament of Treitz (Fig. 4E). The inferior vena cava (IVC) was identified after performing an anti-Kocher maneuver. A piece of gauze was put in the ventral side of IVC and used as a good landmark for the safe dissection between pancreas head and IVC. Then the pancreatic neck was transected with ultrasonic scalpel and the main pancreatic duct was transected with cold scissor (Fig. 4F). Then the SMV and portal vein (PV) were hanged with rubber band and retracted to the left. The superior posterior pancreaticoduodenal vein and other small veins from pancreas head to SMV/PV were transected. The uncinate process of pancreas was dissected at the right side of superior mesenteric artery (SMA) (Fig. 4G). Then the specimen was retracted to right upper quadrant of abdomen and anti-Kocher maneuver was carried out (Fig. 4H). The specimen was resected (Fig. 4I) and put into a retrieval bag and removed from the enlarged umbilicus incision. In terms of gastrointestinal reconstruction, we performed duct-to-mucosa pancreaticojejunostomy for every patient in this series. The details of pancreaticoduodenectomy were described in our previous study [6]. The end to side hepaticojejunostomy was performed with 4-0 monocril. The duodenojejunostomy or gastroenterostomy was performed 45 cm from hepaticojejunostomosis.

### Definitions

A 0 mm margin-free clearance was regarded as R0 resection. Pancreatic fistula was defined as the 2016 update of the International Study Group (ISGPS) definition. Delayed gastric emptying was defined as gastric stasis requiring nasogastric intubation for more than 7 days or the reinsertion of a nasogastric tube. Mortality was defined as any death that directly or indirectly associated with operation within 90 days of surgery.

### Statistical analysis

Statistical analyses were performed using SPSS 16.0 for Windows (IBM, Armonk, NY, USA). Numerical data were expressed as mean ± standard deviation. Differences between variables were compared using Student’s t test, chi-square test, or Fisher’s exact test. Data were considered significant at p < 0.05.

## Results

The demographic characteristics of these patients were shown in Table 1. There were five male patients and eight female patients included in this study. The mean age of these patients was 52.7 ± 14.5 years. The mean body mass index was 23.5 ± 3.2 kg/m^2^. The pathologic diagnosis included four cases of pancreatic intraductal papillary mucinous tumors, two cases of solid pseudopapillary tumors, two cases of duodenal gastrointestinal stromal tumors, two cases of pancreatic neuroendocrine tumors, one case of mucinous cystadenoma, one case of pancreatic ductal adenocarcinoma and one case of pancreatic pseudocyst. Compared with patients in the group 1, more patients in the group 2 suffered from more pancreatic ductal adenocarcinoma, distal bile duct carcinoma and duodenal papillary carcinoma. The size of tumor in the group 1 was significantly larger than that in the group 2(7.2 ± 2.9 cm vs. 2.4 ± 1.7 cm, p < 0.01).

**Table 1:** Demographic characteristics of patients

Variables	Group 1	Group 2	P value
No. of patients	13	141	–
Male/female	5/8	75/66	NS
Age (years)	52.7 ± 14.5	61.8 ± 10.2	NS
BMI (kg/m^2^)	23.5 ± 3.2	22.4 ± 2.7	NS
Tumor size (cm)	7.2 ± 2.9	2.4 ± 1.7	< 0.01
Pathological diagnosis			< 0.01
IPMN	4	15	
SPT	2	8	
PNET	2	6	
DGST	2	3	
PDAC	1	27	
Mucinous cystadenoma	1	4	
Pancreatic pseudocyst	1	2	
DBDC	0	31	
DPC	0	37	
DA	0	5	
Others	0	3	

*BMI* Body mass index, *IPMN* Intraductal papillary mucinous neoplasm, *SPT* Pancreatic solid pseudopapillary tumors, *PNET* Pancreatic neuroendocrine tumors, *DGST* Duodenal gastrointestinal stromal tumors, *PDAC* Pancreatic ductal adenocarcinoma, *DBDC* Distal bile duct carcinoma, *DPC* Duodenal papillary carcinoma, *DA* Duodenal adenocarcinoma, *NS* not significant

The operative details and post-operative outcomes were shown in Table 2. One patient in the group 1 converted to open surgery because of uncontrollable hemorrhage, which caused by serious adhesion between tumor and SMV. Seven patients underwent laparoscopic pylorus-persevering pancreaticoduodenectomy and six patients underwent LPD. The patients in the group 1 required longer operative time (356 ± 47 min vs. 312 ± 36 min, p = 0.02) and suffered more blood loss (325 ± 216 ml vs. 168 ± 72 ml, p < 0.01). Three patients in the group 1 required blood transfusion. Two patients required blood transfusion because of severe anemia caused by recurrent gastrointestinal bleeding. Only one patient required blood transfusion due to intra-operative bleeding. The R0 rate and the number of lymph nodes harvested were comparable between two groups. The post-operative outcomes of patients in the group 1 were favorable. Only one patient suffered from grade B pancreatic fistula due to delayed removal of drainage (25 days). One patient suffered from delayed gastric emptying which was cured by conservative therapy. No patient suffered from post-operative abdominal bleeding or abdominal abscess. No patient required re-operation or percutaneous drainage. 90-day mortality of patients in the group 1 was zero. The mean post-operative hospital stay, overall complication and pancreatic fistula rate were comparable between two groups.

**Table 2:** Operative and post-operative outcomes

Variables	Group 1	Group 2	P value
Operative time (min)	356 ± 47	312 ± 36	0.02
EBL (ml)	325 ± 216	168 ± 72	< 0.01
Conversion (n, %)	1, 7.7%	3, 2.1%	NS
Transfusion (n, %)	3, 23.1%	7, 5.0%	0.04
R0 resection (n, %)	13, 100%	139, 98.6%	NS
Lymph nodes harvested	15.8 ± 2.6	17.2 ± 3.5	NS
POHS (days)	12.4 ± 1.9	12.1 ± 2.3	NS
Complications (n, %)			NS
Pancreatic fistula			
Grade B	1, 7.7%	12, 8.5%	
Grade C	0	1, 0.7%	
DGE	1, 7.7%	7, 5.0%	
Abdominal bleeding	0	1, 0.7%	
Bile leakage	0	4, 2.8%	
Chyle leakage	0	5, 3.5%	
Abdominal abscess	0	2, 1.4%	
Re-operation	0	3, 2.1%	NS
90-days mortality	0	1, 0.7%	NS

*EBL* Estimated blood loss, *POHS* post-operative hospital stay, *DGE* delayed gastric emptying, *NS* not significant

## Discussion

Although firstly reported in 1994 by Gagner [7], LPD is still one of the most challenging minimal invasive abdominal surgeries. LPD has not yet been widely adopted in the first two decades due to there was no clear evidence in favor of LPD over open pancreaticoduodenectomy (OPD) in terms of operative time, blood loss, length of stay or rate of complications. However, LPD is beginning to gain wider acceptance in the past decade owing to the accumulation of surgical experience and evolution in laparoscopic technology. Many systematic reviews shown that LPD was associated with less intraoperative blood loss and postoperative morbidity and may serve as a promising alternative to OPD in selected patients [7–10].

However, Zhang et al. found that the tumor size in the LPD group was smaller than that in OPD group in their meta-analysis [9]. Periampullary tumors larger that 5 cm was considered as a relative contraindication for LPD in many institutions. Kocher maneuver was a standard or the conventional approach during PD. This approach was considered to be essential in reducing blood loss. However, it is difficult to perform Kocher maneuver in patients with periampullary tumor larger than 5 cm due to limited space during LPD. Injudicious Kocher maneuver in such cases may lead to excessive bleeding caused by avulsion of the veins around tumors, iatrogenic tumor rupture, and spillage of cancer cells into portal system. To date, LPD without Kocher maneuver has not been reported in the literature.

The anterior approach technique was well documented in open right hepatectomy for large tumors [12]. This technique involves initial inflow control, completely parenchymal transection and outflow control, before the right liver mobilization [13]. Many studies found that the anterior approach in right hepatectomy resulted in better operative and survival outcomes compared with the conventional approach [5]. We found that there were many similarities between the anterior approach LPD and the anterior approach laparoscopic right hepatectomy for large tumors. In order to perform in situ resection and decrease blood loss, we also carried out vascular inflow control (gastroduodenal artery, right gastric artery, and right gastroepiploic artery) before pancreas neck dissection. After pancreas neck transection, the veins from pancreas head to PV/SMV(outflow control)could be shown and dissected before uncinate process resection. Kocher maneuver was carried out after completely inflow control, pancreas parenchymal transection and outflow control. Although patients in the group 1 suffered more blood loss due to bleeding from peritumoral varices, only one patient required blood transfusion due to intraoperative bleeding.

Hematogenous dissemination of malignant tumor cells has been reported during surgical resection of biliary-pancreas cancer and colorectal cancer [14]. According to literature, liver metastasis is one of the most common patterns of periampullary tumor recurrence [15]. LPD via anterior approach may provide theoretical oncological advantages over conventional approach. The pancreas head together with the tumor could be completely separated from the PV/SMV before mobilization. This is a kind of “no touch technique”, which can avoid squeezing the tumor cells into the portal vein. However, the long-term outcomes of this technique for malignant tumors require further investigation.

It should be noted that there were several limitations associated with this technique. Firstly, it is technique more challenging than LPD via conventional approach. We just apply this approach in patients with very large periampullary tumors. The indications for this approach will gradually expand in the future. Secondly, we could not make sufficient intra-operative evaluation of tumor invasion to inferior vena cava or superior mesenteric artery due to the absence of Kocher maneuver. Due to its high invasiveness, pancreatic ductal adenocarcinoma (PDAC) larger than 5 cm is often associated with vascular invasion or metastasis. Accordingly, it is very important to perform fully pre-operative evaluation of resectability of tumor, especially for the PDAC. Fortunately, radiological resectability assessment could provide accurate evaluation of tumor resectability [16, 17]. We routinely performed three-dimensional computed tomography of upper abdominal vessels. The resectability was accurately evaluated in all cases in present study. We do not recommend this technique for patients with suspected inferior vena cava or superior mesenteric artery involvement. Thirdly, the long-term oncological outcomes were absent in this study. The oncological benefits of this approach require further investigation.

## Conclusions

Laparoscopic pancreaticoduodenectomy via anterior approach is safe and feasible for patients with large periampullary tumors. Its oncological benefit requires further investigation.

## Data Availability

All data generated or analysed during this study are included in this published article.

## Abbreviations

LPD: Laparoscopic pancreaticoduodenectomy
OPD: Open pancreaticoduodenectomy
SMV: Superior mesenteric vein
SMA: Superior mesenteric artery
PV: Portal vein
GDA: Gastroduodenal artery
IVC: Inferior vena cava
CHA: Common hepatic artery
